# INVESTIGATING MALE INFERTILITY: EDITING GENES IMPORTANT FOR SPERMATOGENESIS IN A CELL CULTURE SYSTEM

**DOI:** 10.1093/geroni/igac059.2939

**Published:** 2022-12-20

**Authors:** Cassandra Norbeck, Kellie Agrimson

**Affiliations:** Saint Catherine University, Saint Paul, Minnesota, United States; Saint Catherine University, Saint Paul, Minnesota, United States

## Abstract

The four levels of aging include the specific disease, the systemic complications that lead to the disease, the loss of cellular integrity that affects the system, and macromolecule malfunction that alters cellular integrity. Similarly, this project investigates infertility and testicular cancer, misregulation of the endocrine system, problems with germ and Sertoli cell maintenance, and ultimately gene expression changes that alter cellular proliferation and death. Infertility affects 4.5–6% of North American males and up to 15% of couples worldwide. Infertility may be caused by unknown genetic factors, as up to 2,300 genes are pertinent to male fertility. This research project aims to create the molecular toolbox needed to evaluate gene function in cultured male germ cells. We will use mouse primary spermatogonial stem cells and a human testicular cancer cell line to knock out the functions of genes Phosphoprotein 1 (Spp1) and Inhibitor of DNA Binding 4 (Id4) in the testis to determine the effect of the knockouts on male germ cell proliferation and cell death. We designed single guide RNAs (gRNAs) using online bioinformatic tools and amplified the genes from human and mouse genomic DNA to demonstrate the effectiveness of our gRNAs in vitro. These gRNAs and Cas9 compose the beginnings of the molecular toolbox used to electroporate cultured cells. This research will contribute to the greater scientific community by providing insight into the function of Spp1 and Id4 relating to male fertility and providing a methodology for future research in aging-related fertility diseases and testicular cancer.

